# Recueil de Memoires de Medecine, de Chirurgie, et de Pharmacie Militaires. Vol. LXII

**Published:** 1847-07-01

**Authors:** 


### Recueil de Memoires de Medecine, de Chirurgie, et de Pharmacie Militaires. Vol. LXII. Octavo, pp. 400. Paris, 1846.

In availing ourselves of the contents of the successive volumes of this "Recueil," issued under the superintendence of a Committee of the Council of Health of the French Army, which selects from the various documents forwarded by the medical officers of the service those that it deems most fitting for publication, we have often regretted that our own military medical authorities have not, in a similar manner, contributed to the diffusion of the ample and valuable materials which have been, from time to time, deposited in such abundance in the archives of the war department. Something of the kind has been done of late by the publication of the Army and Navy Medical Statistical Reports; and it is to be hoped that the universal approbation bestowed upon these valuable works will incite those in power to continued analogous undertakings.

The present volume contains some interesting papers, which we proceed to notice.

### I. Fragments from the Surgical Clinic of M. *Sedillot,* Senior Surgeon to the Hospital of Instruction at Strasburg.

1. *Purulent Infection.*—M. Sedillot believes that authors have too generally regarded this affection as constantly fatal in consequence of their only taking into consideration extreme cases. He establishes a distinction between *purulent infection* and *metastatic abscesses.* As long as the disease is confined to the former condition it may be cured; if there are abscesses only of small size, or few in number, all hope is not extinct; death only being inevitable when these are very numerous or large, or open into the pleura, the articulations, &c. The effects vary much also, not only according to the quantity of pus mingled with the blood, but also according to its qualities—the pus from a phlegmon producing much less deleterious effect than a sanious pus. Wounds of the perineum, in which there is a mixture of pus and urine, produce, even when the suppuration is not very abundant, fatal effects in a very brief space of time. It may be replied to the statement that the less advanced cases of purulent affection recover, that such were not examples of the disease at all; but M. Sedillot believes the pathological changes induced in man and animals from this cause are the same, and numerous experiments upon these last have proved to him—1. That a small quantity of pus injected into the veins only produces slight effects. 2. If the injection be repeated for several successive days, thirst, shivering, &c. are produced; but the animal continues to live if they are then discontinued—so that we must kill it in order to observe the pathological alterations at this period, such as patches in the lungs, emphysema, &c. 3. If a new portion of pus be daily injected, death takes place, always producing the same changes.

The lungs are the organs in which pus is found to be most frequently deposited in this affection; then follow the pleuræ, the joints, the liver, and the muscles. Although veins are constantly found leading from the source of pus, in a great number of cases no trace of *phlebitis* is visible. After amputations, in deep-seated phlegmons, in chronic suppuration, caries, &c., it is always by means of the divided or eroded veins that a direct communication between the purulent centre and the circulation is established, and the mixture of pus with blood which this gives rise to is one of the best ascertained phenomena of the disease. The constant obliteration of the veins by coagula, even in the cases in which they are inflamed, is, contrary to the statement of most authors, an exceptional occurrence. The coagulum, when it exists, does not adhere to the walls of the vein, but floats in the pus, having an elongated, fusiform, shape. If it is interrupted from place to place, the blood remains fluid in the intervals, having lost its red colour, and become converted into a sanies by admixture with pus.

Recognizing different stages of this affection, and its curability in some of these, M. Sedillot enumerates the following indications of treatment. 1. Combating the inflammatory symptoms, if intense, by bleeding, especially local. 2. Modifying the surface secreting the pus, in the case of a wound. This is to be done by stimulant lotions or baths, or injections of aromatic wine. In this way the vitality of the tissues becomes modified, and the pus changed in qualities, or its secretion arrested. 3. Furnishing ample exit for pus by prompt incisions if necessary. 4. The frequent renewal of dressings. 5. The use of the actual cautery. This is often very efficacious. 6. If purulent infection seems threatened after attempting union by the first intention, the commencing cicatrix is to be broken, and the edges of the solution of continuity irritated. 7. A revulsive action of the secretory organs is to be maintained, especially by the use of purgatives. 8. Cold fluids should be drank in abundance, to maintain the venous system in a state of repletion, and diminish its absorbing powers as much as possible. 9. Counter-irritants should be applied in the vicinity of any organs suffering from derangement of function. 10. Tonics are not indicated until the febrile action has declined, and true prostration set in. 11. In the case of symptoms of infection occurring in a carious limb, amputation offers the best resource if its performance be not too long delayed.

2. *Union by the First Intention.*—In a section upon extirpation of the cervical glands, M. Sedillot takes occasion to express his opinion upon the union of wounds by the first intention. He is not an advocate in general for attempting this, as, in the hands of himself and the other surgeons of Strasburg, it seldom succeeds. When it is incomplete, the inconveniences it gives rise to are not compensated by its advantages, and the wound is healed more certainly, and nearly as rapidly, by dressing it with a barred compress and lint. When immediate union occurs, sometimes the integuments are alone agglutinated, the deeper portions of the wound not having undergone reparation. By the other method, the healing takes place from within outwards. In determining the question, *climate* must be taken into consideration. In the high temperature and dry air of Egypt union by the first intention produces wonderful results, and M. Serres has observed the same thing to some extent at Montpellier: but in cold and damp climates, like those of Paris and Strasburg, such a mode of union is only the exceptional case. Alluding to the same subject, when treating of amputation of the thigh, M. Sedillot adds:—

"The most exact approximation cannot prevent an excavation which serves as a reservoir for the fluids exuding from the wound. A pultaceous, greyish matter, a species of false membrane formed of the fibrous portion of the blood, which should be eliminated, is retained within the wound, and converted into a purulent and fetid sanies, which may give rise to severe consequences, such as phlebitis, purulent infection, &c. The bone, macerated in the pus, becomes denuded of the periosteum and necrosed. All this while adhesion of the skin, which is easily produced, masks what is going on in deep-seated parts until tension, tumefaction, and heat reveal it. Unless incisions are practised, or the cicatrization torn open, collections of matter are formed, and the *morale* of the patient, who believing himself nearly cured finds that all is well nigh to suffer again, becomes injuriously influenced.

"More security attends a different practice. After the ligature of the vessels the blood is to be carefully staunched, and the wound is neither to be closed by strapping or crammed with lint. A barred compress spread with cerate, and a few pieces of fine lint, are alone to be applied, the muscles of the limb being supported by a roller. During the first days the surface of the wound becomes covered with a greyish, pultaceous crust, which however is soon detached, leaving red, healthy granulations—the precursors of cicatrization. The fears of the patient of the first dressing have been especially objected to his plan: but such objection is only valid when the dressing is performed prior to the complete establishment of suppuration, and ceases to be so if this be delayed to the fourth or fifth day, when the compresses are nearly detached by the suppuration, and their removal gives relief instead of pain to the patient. After this the wound should be dressed daily, for frequent dressings are the best means for the prevention of danger from the sojourn of pus."

For the union of wounds by means of *pins, needles,* or the *twisted suture,* M. Sedillot gives the following precepts.

"When the solution of continuity is not very considerable, two or three points of suture are sufficient, as strips of plaster may be applied along the rest of its course. Surgical incisions or accidental wounds of more than certain extent require a greater number. In this case we should apply the first one at about the middle of the incision, as we can in this way judge much better of the disposition of the two lips of the wound, and remedy any imperfections or defects of relation. Before passing the needle we must smear its point with grease, taking especial care to free our fingers from this afterwards. When we wish to withdraw it, we should carefully free its point from all matters which can impede its exit, support the edges of the wound by our fingers while removing it, and leave in the threads for some days longer. The third or fourth day is generally the proper period for the removal of the needles, unless severe pain, tension, redness of the edges of the wound, or inflammation accompanied by fever, occur. In these cases we must remove them at once, and combat the accidents by appropriate measures."

3. *Extirpation of the Amygdalæ.*—The humid climate of Strasburg seems very favourable for the production of angina and consequent tonsillar hypertrophy, for in one year M. Sedillot performed the operation of extirpation no less than thirty times. The operation may be performed by means of Fahnestock's amygdalotome, or by the bistoury. The former, requiring the aid of no other instrument, simplifies the operation, frightens the patients much less, and causes less pain: but it is a bad instrument, as only a part of the structure is removed, and resolution very rarely follows. The straight, probe-pointed, narrow-bladed bistoury, protected by a piece of linen to within a certain distance from its extremity, is the best one, requiring however the assistance of a hook or forceps. The section should be made from below upwards, with the double object of not covering with blood the parts yet to be divided, and being able, in case of need, to suspend the operation before complete section, the divided portion still held by its upper segment not being then liable to produce mischief by falling back upon the glottis. In operating upon children, a difficulty presents itself in keeping the mouth open. Cork is usually used for this purpose; but if it is too small it is easily displaced, and if too large it takes up too much space. M. Sedillot employs with advantage gold or silver *thimbles,* by which an assistant guards his fingers, and is then easily enabled to keep the mouth open as widely as may be required.

### II. Several Cases of Congelation. By Dr. *Shrimpton.*

An episode in the Algerian Campaign of 1845-6 furnishes the material for this paper. During the retreat of a column of the army (its numbers are not stated) from Bou-Thaleb in January, its progress became arrested by heavy snows for two or three days, and, provisions falling short, above 200 men died on the route. The particular detachment which Dr. S. accompanied lost no one, nor did any one in it suffer *en route* from congelation, which results, he attributes, to the beneficial effect of a few drops of brandy which he was twice enabled to distribute with some bread, and to the circumstance of the men when lying down keeping huddled close together. Although a strong north wind blew the cold was by no means intense enough to account for the large mortality. Prior to the retreat, too, the labours of the soldiers had not been excessive, nor had they been subjected to any privations. The great scarcity of food, and the fact of some of the most robust perishing, seem to indicate that defective alimentation was the cause of these results; man requiring an energetic nutrition to resist the influence of low temperatures. The phenomena accompanying death by congelation, Dr. S. thus describes :

"I have frequently been a witness of the symptoms which precede death from congelation. The persons experience a general sense of stiffness or numbness, accompanied sometimes with pains in the limbs and groins. Muscular contraction is performed with difficulty. The face becomes red and swollen, the lips blueish, the eyes projecting, and the hands red and tumid. The pulse is small and feeble and the respiration slow. All these symptoms rapidly increase. The eyes then take on a wandering expression, the step is uncertain and vacillating, and at last the man falls down. The skin of the hands cracks and frequently gives issue to from two to four ounces of blood. The patient preserves his senses, but has all the appearance of a drunken man. If he is raised up he falls down again, and he cannot be kept in a litter without being tied to it."

Upon the arrival of the column at Setif, 532 patients were admitted into the hospital on account of the effects of congelation. Of these, 186

were cases in which phlyctenæ or slight ulcerations followed congestion of the feet or hands, 60 in which the loss of one or more of the nails occurred, and 221 in which superficial gangrene took place without implicating the bones or joints. In 52 cases, in which these latter parts became affected, amputation was performed, three of the patients only dying after the operation, although the wounds for some days put on a very flabby aspect, and were in some parts disposed to slough. Eleven cases perished from sphacelus of the extremeties, or consecutive internal lesions. The feet, as might be expected, from their constant exposure to melting snow, suffered in the vast majority of cases; and thus, of 532 cases, in only 26 was there congelation of the hands. The *local* effects of congelation are strikingly *identical with those produced by burning* in its various degrees; so much so, indeed, that the one case could not by mere inspection have been distinguished from the other. In the fatal cases, marked pulmonary and cerebral congestions, or even inflammation, were found, and, as in severe cases of burns, gastro-intestinal irritation was set up, which sometimes proceeded to ulceration of the ileum and colon. The local inflammatory re-action after congelation was, however, less prompt and marked than after burns, and was often absent, giving rise to extensive lesions after the fall of the eschars. On the other hand, parts which were even blue, or black, from local asphyxia, sometimes promptly recovered their normal condition under the influence of this re-action.

In respect to *treatment*, the patients who seemed in danger of asphyxia, were enveloped in bed-covering, while some hot broth was given them. Very soon after this they fell asleep, and a mild re-action generally became established. In some cases detailed, fatal inflammation of the lungs or membranes of the brain was developed. The milder local effects were generally cured in from four to eighteen days, pains however persisting in the joints of the hands and feet for one or several months. The wounds were simply dressed and the limbs enveloped in flannel moistened with camphorated oil. The benumbed parts were gently rubbed until re-action became established. The phlyctenæ were preserved as long as possible, especially in the slighter cases, and, when the parts were much engorged, incisions were practised in order to prevent the spread of gangrene. Warm fomentations and poultices were also employed during the first days. All those who were enabled to leave their beds were encouraged to do so and go into the airing courts, while the wards, bedding, &c. were thoroughly cleansed and ventilated—a precaution rendered essential by the crowded state of the hospital, 528 patients occupying a space intended only for 200. Substantial diet and, as soon as possible, wine were prescribed.

### III. On the early Use of Injections in Gonorrhœa. By Dr. *Poullain.*

The employment of injections as an abortive mode of treating gonorrhœa, a practice never received with much favour in this country, has recently re-excited great attention in France. M. Poullain, from his position of Surgeon to the Military Hospital at Lyons, has had great opportunities of investigating the practice, and in the present very able paper details the results he has obtained.

Regarding gonorrhœa as a *specific* inflammation of the urethra (*blenno-urethritis*), he believes our object should be to destroy this "*specificity*" and reduce the disease to the condition of an ordinary or "normal inflammation." This he believes is best accomplished by the prompt use of injections. He animadverts upon the opinion advocated by Baumès and others, that the sudden suppression of the discharge in this way is sometimes followed by the outbreak of *secondary symptoms.* In 600 patients treated by the abortive method by Dr. Poullain, during the last fifteen years, he has never met with an instance of this, and in 200 other cases, whose subsequent history he has been in the position to assure himself of, not one occurred. He fully agrees with M. Ricord, as we believe most other observers now do, that the apparent examples of constitutional symptoms following gonorrhœa, are really examples of *concealed urethral chancre.* To this opinion it has been objected, by Baumès and others, that the chancrous matter applied at the orifice of the urethra can scarcely be expected to travel to the prostatic portion before producing its local effects. But these observers are guilty of the inconsistency of attributing this very power to gonorrhœal matter when they state the vicinity of this portion of the urethra to be its commonest source. But the possibility is best decided by facts. A soldier who had been long fruitlessly treated for gonorrhœa was carried off by typhus, and, on examining the urethra, a well-characterized chancre was found at 10 or 12 lines behind the *fossa navicularis.* In two other cases of gonorrhœal patients, dying of other diseases, well marked cicatrices of venereal ulcers were found at nearly three inches from the orifice.

As in all other inflammations the prevention of the formation of a *chronic* stage, and the consequent pathological changes in the parts implicated, is highly important, and M. P. believes this has not hitherto been accomplished by injections because they have been employed too tardily. Reversing the ordinary procedure, he always uses them in the *acute stage, rarely in the chronic one;* and it is their having been confined to this last that has obtained for them the ill-reputation of producing various ill-effects which really result from the disease having already passed into a chronic condition. The object in recommending early injection is not to procure the cessation of the discharge, but a change in the character of the inflammation; for, this once accomplished, as the diminution of pain and other symptoms prove it to be, the discharge usually soon disappears of itself, or is easily removeable by means of balsams and revulsives. The prejudice against their use, founded upon their supposed tendency to the production of *stricture,* is a very erroneous one; for we cannot suppose this to be formed without the prior existence of inflammation.

In fact, they are the best means for the prevention of this affection. If stricture arises from the use of injections it ought to be formed at the various portions of the canal the stimulating fluid comes in contact with; but in the vast majority of cases it is so in a very limited portion of the canal and one to which the injection hardly ever obtains access. If, too, we question persons suffering from stricture we find that they have rarely made use of injections, and that they have found the canal become narrowed only after protracted duration of the discharge. Injections are indicated then during the whole of the acute stage, and no other means is so efficacious for calming the ardor urinæ, and for relieving painful erections. All these symptoms, however violent they may be, yield, as if by enchantment, to the use of injections, and, so far from contra-indicating them, form the strongest argument for their employment. " In this way I constantly arrest numbers of gonorrhœas without any ill-effects resulting." Dr. Poullain employs the following modification of Bell's formula—*Sulph. Zinci, a scruple, Aq. Destil.* 10 *oz.* Dissolve and add *Liq. Plumb. Acetat. gtt.* 20, *Laudanum of Sydenham* 1 *drachm.* The last ingredient he regards as an important one. This injection is to be employed as soon as slight irritation, redness, or discharge is seen, the sooner the better. Three jets should be thrown in three or four times daily, and the fluid retained in the canal by slight pressure for half a minute. In most cases the pain and inflammation disappear in 24 hours, or at farthest in a day or two, and the patients often are quite surprised at the rapidity of the relief. The injections must, however, be continued until the discharge becomes sparing and colourless, which generally occurs in about from five to ten days. They may be then discontinued as the discharge soon ceases, or may be removed by ordinary means. Although injections act most beneficially when employed at the very commencement, they are still useful at a more advanced stage, and we must not allow the severity of the symptoms to prevent our employing them, as they will promptly alleviate these. The eight or ten first days constitute the most eligible period for their use, but we may still have recourse to them at the fifteenth or twenty-fifth day, when however their success becomes less prompt and less complete; and, although they may be used without danger, they do not always then arrest the discharge; but require the aid of balsams or cubebs administered internally.

*Gleet.*—The means advocated does not require any especial abstinence, save from wines or spirits, &c., and will, as a general rule, effect a cure in fifteen days, and frequently in ten, five, or fewer still. When injections have been delayed until the decline of the acute stage the discharge may persist under the form of *gleet* or military gout. The persistence of gleety discharge seems dependent upon four different conditions of the urethral mucous membrane. 1. A simple state of local relaxation. 2. A superficial but circumscribed ulceration. 3. A concealed chancre; and 4. A commencing or already-formed stricture. The first of these is the most common variety, and generally arises from an atonic state of the exhalent vessels of the urethra, consequent upon a condition of excitement which had been treated by demulcents, antiphlogistics, and diuretics. In such cases, *tonic* rather than astringent *injections* best succeed, and Dr. P. usually employs aromatic wine, diluted with equal parts of water, or cold water to which a sixth part of alcohol has been added. In some cases, cold lotions to the penis and scrotum succeed. This variety of gleet always exists without pain, even during the passage of urine or instruments, or the presence of erections. The discharge varies in appearance, according to the manner it has been already treated. It is opaque, milky, or yellowish if it has been left to itself, or what is nearly the same thing, treated by demulcents. It is clear and transparent if injections have been employed. Contagious in the first case, it is never so in the second—an additional advantage for the abortive method. (An advantage, if true, which is not the case.—*Rev.*)

(2.) If this discharge persists for a certain period the portion of the mucous membrane wherein it originates becomes *excoriated*, constituting the second variety, which may always be easily recognized by the change in colour of the discharge, which becomes a dirty white or sanguinolent. In urining too, the patient experiences a sharp pain at the excoriated spot, and he is much tormented by nocturnal erections. In this case, the *nitrate of silver* (1 to 2 grains to the ounce of water) forms the best injection, throwing it in once daily, while in other cases, much success attends its use in the solid form. The discharge, however, after disappearing, returns again with the greatest ease, and upon the slightest excess: and in such cases, a large blister applied to the upper and inner part of the thigh, kept open for a fortnight or even a month, rarely fails in entirely suppressing it.

The *third* variety depends upon a *concealed chancre*. The author relates two remarkable cases of gleet which, obstinately resisting all other modes of treatment, yielded readily to the use of mercury, which such obstinacy and the local suffering induced him to administer. In this way he explains similar examples of the utility of mercury in gonorrhœa recorded by various authors. In consequence of the passage of the urine, the cure of a chancre, under such circumstances, is always tedious and difficult; and even those which are situated near the orifice of the urethra are months in healing, protect them how we may. The diagnosis of a concealed chancre is usually obscure, and we must much depend upon the prior history of the case and upon the presence of certain symptoms, especially an almost constant pain at one point of the canal, increased by urining, by external pressure, or the passage of an instrument. Erections, too, are often as frequent and as painful as in the acute stage of the disease. The discharge is of a sanious and purulent character, sometimes sanguinolent, and, after urining, drops of pure blood may be passed.

The *fourth* variety, that dependent upon stricture, is to be relieved only by the cure of the latter. Stricture, as a consequence of venereal affection, may form in two modes. In the first, the canal in one or more places is obstructed by a kind of vegetation or fleshy excrescence, which M. P. has often met with in opening the urethra after death, sometimes disposed in a parallel direction to the canal, and at others forming a transverse bridle. Upon the passage of an instrument, during life, these are sometimes detected by the readiness with which they bleed. This species of stricture is neither the most common or the most difficult of cure. The removal of the vegetations by cauterization, and the subsequent employment of mercury constitute the treatment. The most common variety of stricture is formed very gradually, and the calibre of the canal is narrowed, generally just anterior to the prostatic portion, in consequence of the thickening of its mucous membrane induced by chronic inflammation. There is usually but little pain, unless the narrowing be sufficient to produce great obstruction to the expulsion of urine. Bougies form the appropriate means of treatment, and it is an error to substitute cauterization for them. They must, however, be most perseveringly employed, and allowed to remain in the urethra for a longer or shorter period, or the stricture will soon re-appear.

### IV. Frequency of Tænia in Algeria.

From the Reports furnished to the compilers of the Recueil this parasite seems especially to infest the soldier in Algeria, although the relative degree in which it does so would have been better judged of if the respective numerical amount of soldiers in the different localities had been stated. The military hospitals of the interior of France furnished, during the years 1840—6, only two cases of tænia, and those of Corsica no case; while no less than 34 cases were observed during the same space of time in Algeria.

### V. Report of the Venereal Patients under the Care of M. Marchal (de Calvi), of the Val de Grace. By *M. Souhaut.*

This is an interesting account of the practice pursued by M. Marchal in the cases of between 600 and 700 patients, who passed through his hands in this military hospital during four months of 1846. The doctrines of M. Ricord upon syphilis were put into practical operation to a greater extent than we should have expected or can approve of. For example, the following positions are, in our opinion, full of the most dangerous fallacies.*

"If the chancre continues simple that is not *indurated*, there is no cause to fear general symptoms. To this primary law, laid down by M. Ricord, we have met with no one exception. Suppurating bubo is always coincident with simple chancre, and excludes constitutional syphilis. We might say that the inflammation developed in the gland prevents the virus passing further, and that this is eliminated by the suppuration. We have met with facts which throw doubt over the first portion of this second law. Induration, according to M. Ricord, is the first general or constitutional phenomenon of syphilis."

*Chancre* varies in its form, duration, and nature. In treating of its seat, several cases of *urethral chancre* are detailed. A reddish appearance of the discharge from the canal is much relied upon as a sign of this, added to which, induration may be sometimes felt by external examination, although, in other cases, a simple inflammatory engorgement may be mistaken for this. The two, however, are distinguished by the dispersion of engorgements by suppuration or antiphlogistics. A severe pain at one point in urining, though not pathognomonic of concealed chancre, deserves attention. Chancres attacking the side of the *frænum*, burrow under it until they leave only, as it were, a little bridge, which, containing in its

* For some excellent criticisms upon the views of M. Ricord, as well as for a most masterly estimate of the various debateable points in the history and treatment of this disease, we beg to refer our readers to Mr. Porter's Lectures, publishing in the Dublin Medical Press of the present year.

substance a small arterial branch, remains undestroyed. The duration of the treatment is abridged by the division of this portion, which M. Marchal accomplishes in the manner recommended by M. Ricord. Two threads are, by means of a needle, passed under the frænum, and the posterior one is first tied round this part, giving the patient much pain, which however he does not feel when the other thread is tied. The frænum is divided between these two, and no blood flows. When chancres exist in considerable numbers around the free *margin* of the *prepuce,* a phymosis, sometimes necessitating operation, is often induced. The case is thus expedited, providing the incision can be made at some little distance from the chancre, so that it may cicatrize as a common wound.

In respect to *form,* the chancre is usually circular, or, as in the case of that of the frænum, very elongated. There is every variety as to size; but it is an error to suppose that small chancres are more readily healed. They sometimes seem to require a time inverse to their size. The chancre may be elevated, superficial, or excavated. In respect to this last, there is one form which may be termed the *pigeon's nest chancre,* which is produced when the ulceration, having destroyed the entire thickness of the preputial mucous membrane, has for its basis only the extremely loose cellular tissue which unites this membrane to the external skin. This cellular tissue becomes excavated beyond the borders of the ulcer, giving the pigeon's nest appearance. The same effect is produced by the chancre commencing externally, and destroying the skin of the prepuce down to the cellular tissue. This variety is especially distinguished by its tedious reparative process.

The average period of *duration* of chancre at Val de Grace was 28 days, some healing far sooner, and others much later. Some chancres may, from their rapid disappearance, be well termed *ephemeral,* and yet these possess all the characteristics and virulence of ordinary chancre. These cases may furnish matter for a bubo which does not appear until after the sore is cicatrized, and then may be taken for a primary bubo. These are not the only cases in which bubo may form after cicatrization. Sometimes patients apply a month after their dismissal, having an open bubo without any new infection.

In respect to the *nature* of chancre, as long as it is *not indurated,* it is a mere *local* disease, but no sooner does induration present itself, than a general infection of the system is imminent or acquired. *Simple superficial chancre* may be confounded with *herpes,* but herpetic ulcerations are usually in clusters, are preceded by pruritus, and accompanied by vesicles in some other portion of the prepuce. In M. Marchal's opinion, however, the distinction is of little practical consequence, as general infection does not follow any simple unindurated ulceration, which promptly cicatrizes. In the hospital many chancres were of a *phagedænic* character. M. Ricord describes three varieties of this, the pultaceous or diphtheritic, phagedæna from excess of induration inducing mortification, and phagedæna from excess of inflammation inducing gangrene. It is the first of these that was met with in Val de Grace. This diphtheritic sore, according to M. Ricord, is marked by absence of induration, the aspect and form of hospital gangrene, and great pain and irritability. This is usually correct, but in one case cited of pultaceous chancre, committing great destruction, induration existed. The diphtheritic appearance is given this chancre by its surface being covered with the tissue destroyed by the ulcerative process—resembling the pulpous variety of hospital sore—and better named *pultaceous*. There is, however, a form of simple non-destructive chancre which is covered by a thin white pseudo-membrane, which may be properly termed simple *pseudo-membranous chancre*. As long as this pseudo-membrane continues, cicatrization will not usually take place, but this must not be mistaken for the white pellicle sometimes seen at the circumference of a healing chancre. There were several examples of bleeding or *hæmorrhagic* chancres—usually very tedious in cicatrizing.

*Induration*.—As this is the point upon which M. Ricord's views may be said to hinge, M. Souhaut enters into the subject of induration somewhat elaborately. M. Ricord considers it as an indication that the economy is infected, and that the disease has become constitutional. M. Marchal hardly goes so far as this, believing rather that it is an indication that infection is imminent, unless therapeutical agents are forthwith resorted to. Induration is not always visible, and must be sought for by seizing the base of the sore between the thumb and index finger. It may often be felt in this way when no-wise in relief, giving the sensation of half of a great pea or a semilunar body under the fingers. There is one form which, to the touch, seems like an empty space under the mucous layer, returning upon itself, like a dry leaf, after the pressure is removed. The induration will vary in degree, according to whether mercury has been employed or not. Until it is removed, slight irritation of any kind suffices to re-produce the ulcer.

*Treatment of Chancre by Cauterization*.—Believing that as long as the chancre continues open the danger of general infection of the economy exists, M. Marchal endeavours to bring about a change in its character, and its prompt cicatrization by its destruction by caustic. The *nitrate of silver* is applicable to a great number of cases, but, in others, where it is desirable to act more deeply, the *Vienna paste* is preferable. Employed on the prepuce, however, this acts too deeply, and gives rise to the pigeon-nest chancre. The cauterization must be repeated as long as there is a pultaceous or pseudo-membranous matter re-produced on the surface of the sore. As soon as the sore puts on a healthy rose-coloured aspect, we must cease. If the chancre is phagedænic, the most active cauterization is indicated. When there are chancres within the prepuce producing *phymosis*, M. Marchal injects a strong solution of nitrate of silver (2 parts to 20) between this and the glans, morning and evening, using injections of opiated aromatic wine in the intervals. This relieves the pain, soon enables the prepuce to be drawn back, an operation is thus usually averted, and the painful tedious wound which results avoided. If called to a patient, while the sore is still in the form of vesicle, it will be the surgeon's fault if this ever become a chancre. A layer of Vienna paste is to be applied to it and beyond its edges. Although so useful for the prevention or changing the character of chancre, cauterization sometimes causes adenitis, but this proceeding not from venereal absorption, but from irritation, soon subsides. This however should prevent our employing caustic, when, from the healing disposition in the sore, it is not required. For local application to the chancre, M. Marchal employs aromatic wine alone, united with laudanum, if there is much pain and irritation, which this much assuages, or with rhatany, tannin, iodine, &c., when the sore is atonic, or retains a bad aspect in spite of repeated cauterization. In some stationary chancres, seated on the glans, diachylon plaister has served to hasten the reparative process; but in chancres of the prepuce this may induce infiltration.

The *induration* requires treatment for two reasons, namely, that it may oppose or retard cicatrization, and that it is a sign of general infection. For the first reason it must be treated with the Vienna paste, and, for the second one, by the internal use of mercury. So, too, induration persisting after cicatrization, slight irritation may lead to the reproduction of the sore, and, as it indicates the continuance of the constitutional disease, as a general rule mercury must not be discontinued as long as it is present. Its disappearance, which is sometimes exceedingly slow, is always to be looked upon as a proof of the satisfactory action of the specific.

### *Blenorrhagic Epididymitis* (*Hernia Humoralis.*)

This disease, in the great majority of cases, is an epididymitis; for, of more than 60 examples in Val de Grace, in only two was the testicle itself affected. On examining the scrotum, the enlarged epididymis may usually be easily felt at some point or another, resembling in shape an acorn and its capsule. The testicle, generally placed in front, retains its characteristic softness, and if pressed, providing the compression do not include the epididymis, no suffering is caused, while acute pain is induced if the epididymis be touched. The error of supposing the testis affected arises from the common occurrence of effusion into the tunica vaginalis, which sometimes becomes exceedingly distended.

The *treatment* employed at Val de Grace as a general rule is antiphlogistic. A general bleeding, and from 20 to 30 leeches being resorted to, repeating these latter (and warm cataplasms) once or oftener. Afterwards, mercurial or opiated camphorated liniments are applied, and pills, containing equal parts of hemlock and calomel, given—the action of the mercury on the mouth being prevented by maintaining a lax condition of the bowels by the aid of injections or Seltzer water. The horizontal position and a spare diet are insisted upon. In spite of this treatment, there is usually left an indurated point of the epididymis, which time alone seems competent to disperse. M. Marchal has very frequently put into practice M. Velpeau's plan of evacuating the serum of the tunica vaginalis by means of numerous punctures, and has observed great relief to the pain, and a more prompt resolution of the inflammation, as the consequences. Frequently, however, the fluid is rapidly re-produced. M. Vidal (de Cassis) has proposed the division (*debridement*) of the tunica albuginea in *parenchymatous orchitis*—a variety so rare that in more than 60 cases not one example presented itself. In more than 20 cases M. Marchal tried with great care the plan recommended by M. Songy,* of keeping the scrotum covered with a thick layer of cotton and wool, well supported in a suspen-

* Medico-Chirurgical Review, No. VII. N. S., page 100.

sory against the external ring, by which means, according to its author, the patient is enabled at once to walk about. In three slight cases this succeeded very well. In several, so great pain was created as to require the instant discontinuance of the attempt, and the rest of the cases remained stationary (save some diminution of pain), and required recourse to be had to antiphlogistics. Several patients, treated antiphlogistically, found the cotton, after the acute stage had disappeared, and while employing ointments and liniments, a better application than poultices. The plan is only of service, therefore, in slight cases.

